# Entwicklung einer Riesenbulla unter Spontanatmung durch „patient self-inflicted lung injury“ bei COVID-19-Pneumonie

**DOI:** 10.1007/s00101-021-01072-w

**Published:** 2021-11-22

**Authors:** Nicholas Moellhoff, Philipp Groene, Ludwig Ney, Daniela Hauer

**Affiliations:** 1 Intensivstation ANIS 5, Klinik für Anaesthesiologie, Klinikum der Universität München, LMU München, Nussbaumstraße 20, 80336 München, Deutschland; 2 Abteilung für Hand‑, Plastische und Ästhetische Chirurgie, Klinikum der Universität München, LMU München, München, Deutschland

**Keywords:** COVID-19, ARDS, Bulla, High-Flow-Therapie, „Patient self-inflicted lung injury“, COVID-19, ARDS, Bulla, High-flow therapy, Patient self-inflicted lung injury

## Abstract

SARS-CoV‑2 und die damit assoziierte COVID-19-Erkrankung stellen Gesundheitssysteme weltweit vor große Herausforderungen. Fast täglich werden neue Erkenntnisse zu Diagnostik, Klinik und Therapie der Erkrankung publiziert. Dieser Fallbericht beschreibt den letalen Krankheitsverlauf eines 81-jährigen Patienten ohne pulmonale Vorerkrankungen, der als Komplikation der COVID-19-Pneumonie unter nichtinvasiver High-Flow-Sauerstofftherapie eine Riesenbulla entwickelte. Pathophysiologisch kommen/kommt eine virusbedingte diffuse Zerstörung des Alveolargewebes und/oder die „patient self-inflicted lung injury“ in Betracht.

## Abstract (en)

The outbreak of SARS-CoV‑2 and the associated COVID-19 pandemic pose major challenges to healthcare systems worldwide. New data on diagnosis, clinical presentation and treatment of the disease are published on a daily basis. This case report describes the fatal course of severe COVID-19 pneumonia in an 81-year-old patient with no previous pulmonary disease who developed a giant bulla during non-invasive high-flow oxygen therapy. Virus-induced diffuse destruction of alveolar tissue or patient self-inflicted lung injury (P-SILI) are discussed as possible pathomechanisms. Future studies must determine whether lung-protective mechanical ventilation with high levels of sedation and paralysis to suppress spontaneous respiratory drive and to reduce transpulmonary pressure can prevent structural lung damage induced both by the virus and P‑SILI in COVID-19 patients with ARDS.

## Anamnese

Ein 81-jähriger, normalgewichtiger Mann mit Vorhofflimmern, arterieller Hypertonie und Hypercholesterinämie stellte sich mit seit 9 Tagen bestehender Schwäche und Atemnot in der internistischen Notaufnahme vor. Hustenreiz verneinte der Patient. Er sei Nichtraucher, sportlich aktiv, fahre durchschnittlich über 50 km Fahrrad/Woche und habe keine pulmonalen Grunderkrankungen. Im Familienkreis gab es mehrere COVID-19-Erkrankungen, bei ihm sei allerdings vor 7 Tagen ein PCR-Test auf SARS-CoV‑2 negativ ausgefallen.

## Befund

In der Notaufnahme zeigte der Patient eine schwere respiratorische Partialinsuffizienz mit kompensatorischer Hyperventilation (p_a_O_2_ 56,4 mm Hg unter Sauerstoff mit 4 l/min, p_a_CO_2_ 25,8 mm Hg, Atemfrequenz 22/min). Ein Antigenschnelltest auf SARS-CoV‑2 war positiv. Eine Aufnahme auf die anästhesiologische Intensivstation wurde zur Überwachung und zur respiratorischen Stabilisierung indiziert.

## Diagnose

### Klinische Untersuchung

Der Patient war bei Aufnahme auf die Intensivstation wach und voll orientiert. Bei Spontanatmung unter Sauersoff mit 6 l/min über die Nasenbrille zeigte sich eine periphere Sättigung von 93 %. Beidseits war ein seitengleich verschärftes Atemgeräusch auskultierbar. Der Patient war normoton, normofrequent und zeigte im EKG die vorbeschriebene Arrhythmia absoluta. Das Abdomen war weich, ohne Druckschmerz und mit regelhaften Darmgeräuschen. Der Patient zeigte eine Spontanmiktion und keine peripheren Ödeme.

### Blutgasanalyse, Aufnahmelabor und PCR-Diagnostik

Die arterielle Blutgasanalyse unter 6 l O_2_/min ergab: p_a_O_2_ 80,6 mm Hg; p_a_CO_2_ 27,5 mm Hg; pH 7,5; BE – 0,6 mmol/l; Atemfrequenz 21/min.

Das Aufnahmelabor zeigte Veränderungen im Sinne einer ausgeprägten COVID-19-Erkrankung: Ferritin: 3928 ng/ml, CRP: 21,8 mg/dl, PCT: 0,3 ng/ml, LDH: 405 U/l, Troponin T: 0,014 ng/ml, Leukozyten: 4,41 G/l, Hb: 13,1 g/dl, Lymphozyten: 0,51 G/l, eosinophile Granulozyten: 0,02 G/l, Fibrinogen: > 900 mg/dl, D‑Dimer: 1,2 µg/ml.

Der PCR-Test auf SARS-CoV‑2 bestätigte die Viruserkrankung, zeigte im kombinierten oro-/nasopharyngealen Abstrich mit ca. 7000 Kopien/ml allerdings eine geringe Viruslast.

### High-Resolution-Computertomographie

**Figure 1:**
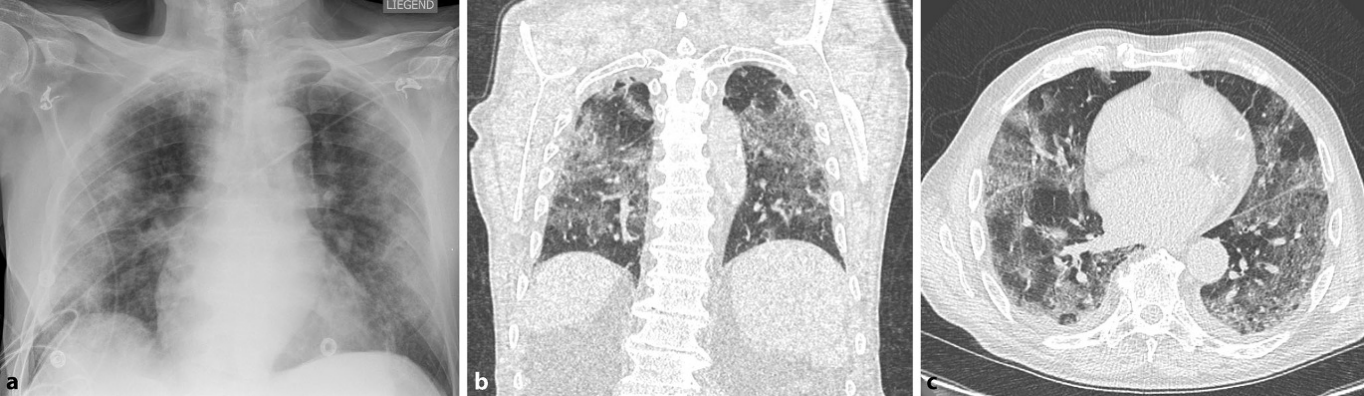

Die CT-Diagnostik zeigte bipulmonale Konsolidierungen sowie Milchglastrübungen, vereinbar mit einer COVID-19-Pneumonie (Abb. 1).

## Therapie und Verlauf

**Figure 2:**
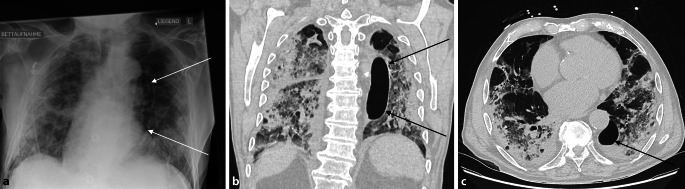

Einen Tag nach Aufnahme wurde bei fortschreitender Hypoxämie eine High-Flow-Sauerstofftherapie (F_I_O_2_ 50 Vol.,%, Flow 40 l/min, darunter: p_a_O_2_ 69,1 mm Hg; p_a_CO_2_ 27,4 mm Hg; pH 7,5; BE −0,1 mmol/l; Atemfrequenz 19/min; Horowitz-Index 138 mm Hg) initiiert, welche im weiteren Verlauf eskaliert wurde. Dyspnoe wurde überwiegend verneint, es zeigte sich aber bereits unter leichter Belastung (Mobilisation an die Bettkante, Atemgymnastik) ein deutlicher Abfall der Sauerstoffsättigung auf 70–80 %. Unter der leitliniengerechten Therapie mit Dexamethason (6 mg/Tag p.o. für 10 Tage) [8] kam es zu einer endogenen Reaktivierung des Herpes-simplex-Virus, die mit Aciclovir behandelt wurde. Aufgrund des bereits verstrichenen Zeitraums seit Symptombeginn war der Therapieversuch mit Remdesivir nicht indiziert. Zum Zeitpunkt des Krankenhausaufenthaltes des Patienten bestand noch keine Empfehlung zur Therapie mit Tocilizumab nach S3-Leitlinie. Der Patient erhielt durchgehend weiterhin seine therapeutische Antikoagulation mit Apixaban. Seit der Aufnahme zeigte der Patient eine übersteigerte Motivation zur Atemgymnastik, mit selbstinitiierten tiefen und häufigen Atembemühungen. Im Röntgenbild zeigte sich 7 Tage nach Aufnahme eine ovale Hypertransparenz paramediastinal links, die sich in einer CT-Untersuchung als neu aufgetretene große Bulla (3,5 × 3,3 × 10,2 cm) ohne Hinweis auf einen Pneumothorax darstellte (Abb. 2). Zu einer spontanen Ruptur der Bulla kam es nicht. Der arterielle Sauerstoffgehalt betrug dauerhaft über 12 ml/dl, dennoch war die respiratorische Insuffizienz trotz Ausschöpfung aller nichtinvasiver Beatmungsmöglichkeiten progredient, sodass am Tag 11 nach Aufnahme die endotracheale Intubation unumgänglich war. Unmittelbar vor Intubation erfolgte ein nichtinvasiver Beatmungsversuch (PEEP 4 mbar; Druckdifferenz 10 mbar, F_I_O_2_ 100 Vol.-%). Die durchgeführte arterielle Blutgasanalyse zeigte die folgenden Parameter: p_a_O_2_ 57,5 mm Hg; p_a_CO_2_ 29,3 mm Hg; pH 7,5; BE 0,7 mmol/l; Tidalvolumen exspiratorisch 1279 ml; Atemfrequenz 29/min; Horowitz-Index 57,5 mm Hg. Der Oxygenierungsindex des Patienten erfüllte damit die Berlin-Kriterien eines schweren COVID-19-assoziierten ARDS. Auch unter der invasiven, druckkontrollierten Beatmung zeigte sich keine Verbesserung des Gasaustausches. Der Patient entwickelte einen rasch progredienten Schock und ein Multiorganversagen (Herz-Kreislauf-Versagen, anurisches Nierenversagen, Laktacidose). Er benötigte hochdosiert kreislaufunterstützende Medikamente (Noradrenalin 2,4 mg/h, Vasopressin 1,5 IE/h, Dobutamin 10 mg/h). Die durchgeführte Herzechographie zeigte einen mittelgradig dilatierten rechten Ventrikel mit eingeschränkter Pumpfunktion, einen leicht hypertrophierten und hypovolämischen linken Ventrikel mit guter Pumpfunktion, keine regionalen Wandbewegungsstörungen, keinen Perikarderguss, keine relevanten Klappenvitien. Eine Bauchlage war aufgrund der Instabilität des Patienten nicht möglich, ebenso wenig qualifizierte sich der Patient für eine ECMO-Therapie. Die Prognose musste aufgrund der bereits vor der akuten Verschlechterung manifesten Lungendestruktionen einerseits und des akuten Schockgeschehens andererseits als infaust eingeschätzt werden. Der Patient verstarb noch am 11. Tag nach Aufnahme.

Die Obduktion ergab einen diffusen Alveolarschaden mit viropathischen Veränderungen sowie multiple subpleurale Bullae sowohl der linken Lunge als auch im rechten Lungenunterlappen. Die größte entsprach der im CT dargestellten Bulla im linken Unterlappen, welche mit 11,1 × 6,0 × 4,2 cm beschrieben wurde, ohne Hinweise auf einen Pneumothorax. Die Todesursache war das respiratorische Versagen bei SARS-CoV-2-Infektion.

## Diskussion

Bullae sind luftgefüllte, dünnwandige Blasen, die durch destruktive Prozesse mit Rarefizierung des Lungenparenchyms entstehen. Sie können primär („vanishing lung syndrome“) oder sekundär im Rahmen eines Lungenemphysems/einer COPD oder anderer Strukturerkrankungen, etwa einer Lungenfibrose, auftreten. Große Bullae können funktionelle Teile der Lunge verdrängen, was zu einer weiteren Verschlechterung des Gasaustausches führt. Eine wesentliche Komplikation ist der Pneumothorax.

Als Folge von COVID-19-Infektionen sind mehrere Fälle von Patienten beschrieben, die unabhängig von pulmonalen Vorerkrankungen Bullae oder konsekutiv Pneumothoraces durch Rupturen entwickelten [1, 6, 7, 9, 11]. Pathophysiologischer Ausgangspunkt ist eine durch SARS-CoV‑2 verursachte diffuse Zerstörung des Alveolargewebes [7], die unter der häufig notwendigen Überdruckbeatmung bei COVID-19-assoziiertem ARDS als „ventilator-induced lung injury“ enorm aggravieren kann [4, 5]. Ob es durch nichtinvasive CPAP-Beatmung zu einer überdruckinduzierten Ausdehnung des durch die COVID-19-Pneumonie geschädigten Lungenparenchyms – und somit zur Bildung von Bullae – kommt, wird ebenfalls diskutiert [1]. In den meisten bislang beschriebenen Fallberichten ging dem Auftreten der Bullae eine (zumindest nichtinvasive) Überdruckbeatmung voraus. Dies stellt aus unserer Sicht ein wesentliches Argument für eine lungenprotektive Beatmung gerade bei COVID-19-Erkrankten dar. Des Weiteren muss eine „patient self-inflicted lung injury“ (P-SILI) diskutiert werden, bei der es durch patienteneigene Atemarbeit und den dadurch verursachten transpulmonalen Druck zu einem Lungenschaden kommt [10]. Ein wesentlicher Treiber der P‑SILI ist der vermehrte Atemantrieb mit erhöhter Atemarbeit und hohen Tidalvolumina. Der dadurch erhöhte transpulmonale Druck kann ein vorbestehendes „capillary leak“ – seinerseits am ehesten eine Folge des Lungenschadens durch die SARS-CoV-2-Infektion – vergrößern und somit das Lungenödem aggravieren [10]. Die NIV-Therapie bei Patienten mit hohem „respiratory drive“ kann noch höhere Tidalvolumina zur Folge haben, und die Kontrolle dieser Volumina ist bei Patienten unter NIV-Therapie stark limitiert [2, 3]. Hohe Tidalvolumina (> 9,5 ml/kgKG) sind dabei prädiktiv für ein NIV-Versagen [3]. Die in diesem Fall beobachteten hohen Tidalvolumina (TV: 1279 ml) während der nichtinvasiven CPAP-Beatmung unmittelbar vor der Intubation unterstreichen dies. In dem hier vorgestellten Fall ist die Lungenparenchymschädigung mit post mortem nachweisbaren viropathischen Veränderungen auf den schweren Verlauf der COVID-19-Pneumonie mit assoziiertem schwerem ARDS (Horowitz-Index 57,5 mm Hg) und HSV-Superinfektion zurückzuführen, eine Beteiligung im Sinne einer P‑SILI als komplizierender Faktor ist zudem wahrscheinlich. Hier entstand die P‑SILI jedoch unter reiner *Spontanatmung ohne Überdruck* unter HFOT, begünstigt durch die exzessive Atemgymnastik mit tiefen und häufigen Atemzügen seitens des Patienten. Hervorzuheben ist zudem die äußerst rapide Entwicklung dieses destruktiven Lungenprozesses, der während des akuten intensivmedizinischen Aufenthaltes, innerhalb von 7 Tagen nach Aufnahme auf die Intensivstation, auftrat.

In der Zukunft muss untersucht werden, ob durch hochprotektive und frühzeitige Ventilation unter Suppression des spontanen Atemantriebs mit minimaler Bewegung des Lungenparenchyms (V_T_ ≤ 4 ml kg^−1^ *und* Bauchlagerung) und verminderter Fluktuation des transpulmonalen Drucks die P‑SILI verhindert und so Lungenstrukturschäden vermieden werden können.

Zusammengefasst unterstreicht der geschilderte Fall, dass es bei einer COVID-19-Pneumonie mit assoziiertem schwerem ARDS bei Patienten ohne pulmonale Grunderkrankung zu strukturellen Umbauprozessen mit der Bildung von Bullae kommen kann. Die Möglichkeit einer Ruptur dieser Bullae wurde bereits in Einzelfällen beschrieben und kann spontan oder durch Überdruckbeatmung auftreten [1, 6, 7, 9, 11]. Bei einer respiratorischen Verschlechterung dieser Patienten sollte differenzialdiagnostisch demnach eine Verdrängung des funktionellen Lungengewebes durch eine Riesenbulla bzw. ein Pneumothorax durch deren Ruptur bedacht werden.

## Fazit für die Praxis

- Schwere Verläufe der COVID-19-Pneumonie können zu Lungenstrukturläsionen mit der Ausbildung von Bullae führen.
- Die CT-Bildgebung ermöglicht die sichere Diagnosestellung.
- Der Zusammenhang zwischen nichtinvasiver CPAP-Beatmung und der Entwicklung von Bullae und Pneumothoraces bei COVID-19-Patienten sollte zukünftig untersucht werden.
- Auch bei Patienten mit Spontanatmung und HFOT können Lungenparenchymschäden entstehen, die durch die P‑SILI aggraviert werden.
